# Phycobilisomes Harbor FNR_L_ in Cyanobacteria

**DOI:** 10.1128/mBio.00669-19

**Published:** 2019-04-23

**Authors:** Haijun Liu, Daniel A. Weisz, Mengru M. Zhang, Ming Cheng, Bojie Zhang, Hao Zhang, Gary S. Gerstenecker, Himadri B. Pakrasi, Michael L. Gross, Robert E. Blankenship

**Affiliations:** a Department of Biology, Washington University in St. Louis, St. Louis, Missouri, USA; b Photosynthetic Antenna Research Center (PARC), Washington University in St. Louis, St. Louis, Missouri, USA; c Department of Chemistry, Washington University in St. Louis, St. Louis, Missouri, USA; University of Hawaii at Manoa; Indiana University Bloomington; University of California, Los Angeles

**Keywords:** CpcL-PBS, isotopic cross-linking, photosynthesis, mass spectrometry

## Abstract

Cyanobacterial phycobilisomes (PBSs) are photosynthetic antenna complexes that harvest light energy and supply it to two reaction centers (RCs) where photochemistry starts. PBSs can be classified into two types, depending on the presence of allophycocyanin (APC): CpcG-PBS and CpcL-PBS. Because the accurate protein composition of CpcL-PBS remains unclear, we describe here its isolation and characterization from the cyanobacterium *Synechocystis* sp. strain 6803. We found that ferredoxin-NADP^+^ oxidoreductase (or FNR_L_), an enzyme involved in both cyclic electron transport and the terminal step of the electron transport chain in oxygenic photosynthesis, is tightly associated with CpcL-PBS as well as with CpcG-PBS. Room temperature and low-temperature fluorescence analyses show a red-shifted emission at 669 nm in CpcL-PBS as a terminal energy emitter without APC. SDS-PAGE and quantitative mass spectrometry reveal an increased content of FNR_L_ and CpcC2, a rod linker protein, in CpcL-PBS compared to that of CpcG-PBS rods, indicative of an elongated CpcL-PBS rod length and its potential functional differences from CpcG-PBS. Furthermore, we combined isotope-encoded cross-linking mass spectrometry with computational protein structure predictions and structural modeling to produce an FNR_L_-PBS binding model that is supported by two cross-links between K^69^ of FNR_L_ and the N terminus of CpcB, one component in PBS, in both CpcG-PBS and CpcL-PBS (cross-link 1), and between the N termini of FNR_L_ and CpcB (cross-link 2). Our data provide a novel functional assembly form of phycobiliproteins and a molecular-level description of the close association of FNR_L_ with phycocyanin in both CpcG-PBS and CpcL-PBS.

## Abstract (precis)

Cyanobacterial light-harvesting complex PBSs are essential for photochemistry in light reactions and for balancing energy flow to carbon fixation in the form of ATP and NADPH. We isolated a new type of PBS without an allophycocyanin core (i.e., CpcL-PBS). CpcL-PBS contains both a spectral red-shifted chromophore, enabling efficient energy transfer to chlorophyll molecules in the reaction centers, and an increased FNR_L_ content with various rod lengths. Identification of a close association of FNR_L_ with both CpcG-PBS and CpcL-PBS brings new insight to its regulatory role for fine-tuning light energy transfer and carbon fixation through both noncyclic and cyclic electron transport.

## INTRODUCTION

Cyanobacteria use phycobilisomes (PBSs) to harvest light energy and to fine-tune energy allocations for the two linked photosystems (1, 2). PBSs are composed of chromophore-associated water-soluble acidic polypeptides called phycobiliproteins (PBPs) and colorless basic subunits called linker proteins. Conventional PBSs consist of a core from which several rod-like subcomplexes protrude (3). Phycocyanin (PC) is the major PBP in the rod and allophycocyanin is the major PBP in the core, with fluorescence emission peaking close to that of chlorophylls in two reaction centers (i.e., photosystem I [PSI] and photosystem II [PSII]). The rod-core linker cyanobacterial phycocyanin protein G (CpcG) plays an important role in the core-containing PBS, the so-called CpcG-PBS. Recently, a new type of PBS was discovered that was specifically associated with tetrameric PSI through a distinct, hydrophobic CpcG variant protein, which was renamed CpcL (4). CpcL-PBS has no allophycocyanin and up to three copies bind at the periphery of the PSI tetramer, indicative of its highly heterogeneous assembly. CpcL-PBS has not been isolated to purity. Thus, the exact protein composition of CpcL-PBS remains unknown. CpcC1 and CpcC2 are two linker proteins connecting hexameric PC, with CpcC1 located in the proximal side of PBS core and CpcC2 in the distal side of PBS core (5).

Light-driven electron transfer in thylakoids results in reduction of the final electron acceptor (NADP^+^) by ferredoxin (Fd). FNR (Fd-NADP^+^ oxidoreductase) in general plays essential roles in regulating cellular redox homeostasis in plants, bacteria, and the mitochondria of eukaryotes (6, 7). In oxygen-evolving photosynthetic organisms, FNR is the last enzyme in the electron transfer chain during photosynthesis from photosystem I to NADP^+^ and provides reducing power for CO_2_ assimilation. The cyanobacterium *Synechocystis* sp. strain PCC 6803 (*Synechocystis* 6803) produces two FNR isoforms: a small FNR similar to that in plant plastids (8, 9) and a large FNR (FNR_L_) associated with the PBSs (10–13) (PBS). FNR_L_ contains three functional domains: a PBS-binding linker domain (FNR_L_-LD), a FAD-binding domain, and a NAD-binding domain, belonging to protein families pfam01383, pfam00667, and pfam00175, respectively. Although FNR_L_ binds to PBS rods, its precise binding site and its function are still unclear (11, 14, 15).

Phylogenetic analysis illustrates the unique evolutionary features of the CpcG/CpcL superfamily (16). In many cyanobacteria, including *Synechocystis* 6803 and *Anabaena* sp. strain PCC 7120 (*Anabaena* 7120), there are two types of PBSs, conventional CpcG-PBS and CpcL-PBS (4, 17–19). CpcG-PBSs from *Synechocystis* 6803 and many other cyanobacteria and red algae have been characterized (3, 16), contrasting with the unsuccessful isolation of CpcL-PBS (17, 20). It is also unclear whether FNR_L_ is associated with CpcL-PBS.

In this study, CpcL-PBS was isolated from *Synechocystis* 6803 and characterized biochemically, spectrally, and structurally. We found that FNR_L_ is associated with CpcL-PBS. Quantitative mass spectrometry (MS) indicated nonstoichiometric binding of FNR_L_ in CpcL-PBS and significant rod length heterogeneity. To accomplish this, we developed a structural proteomic pipeline by combining cross-linking chemistry in combination with liquid chromatography-tandem mass spectrometry (LC-MS/MS) detection of hybrid peptide species and computational biology to pinpoint the structural location of FNR_L_ in both CpcG-PBS and CpcL-PBS.

## RESULTS AND DISCUSSION

### Biochemical preparation and characterization of CpcG-PBS and CpcL-PBS.

**FIG 1:**
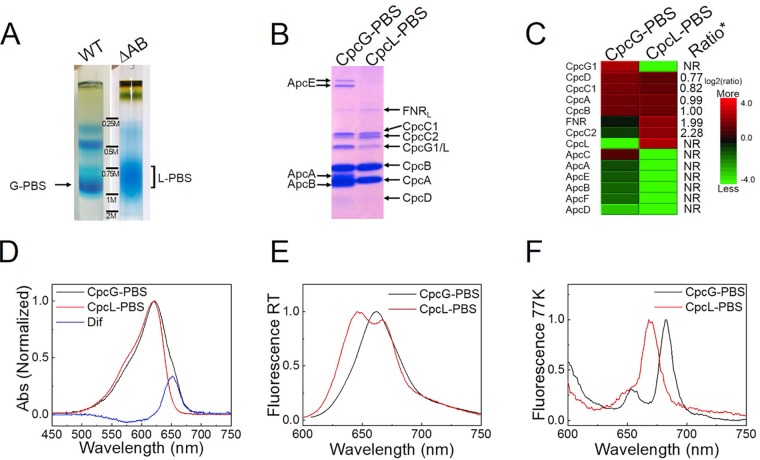
(A) Sucrose density isolation of CpcG-PBS and CpcL-PBS. (B) SDS-PAGE analysis of CpcG-PBS and CpcL-PBS. (C) Mass spectrometry quantification of protein subunits in CpcG-PBS and CpcL-PBS. In the heat map representation, highlighting the component differences, the relevance of effects is related to a ratio, not to a visual difference. Ratio* (PEAKS label-free quantification) indicates the CpcL-PBS/CpcG-PBS ratio. (D) Absorption spectra of the sucrose gradient bands normalized at the absorption maxima. The spectrum difference (Dif) is the difference spectrum between CpcG-PBS and CpcL-PBS. (E and F) Fluorescence emission spectra of CpcG-PBS and CpcL-PBS upon excitation at 580 nm at room temperature (RT) (E) and at 77 K (F).

There are two types of PBSs reported in *Synechocystis* 6803 (17, 21, 22): CpcG-PBS (conventional PBS) and CpcL-PBS (4) (formerly CpcG2-PBS). CpcG-PBS and CpcL-PBS can be successfully fractionated by using step-gradient sucrose density centrifugation of wild-type (WT) and ΔAB (deletion of *apcABC* operon) cells, respectively (Fig. 1A). The ΔAB cells were used because the *apcABC* operon, encoding ApcA, ApcB, and ApcC, was deleted and helped to eliminate any potential ApcA/B protein contaminations to the PC rod preparation. This is superior to the CpcG1 single-deletion mutant cell that still retains large quantities of ApcA/B phycobiliproteins (17). The polypeptide profiles of two PBSs loaded based on a normalized amount of PC (phycocyanin) (23) are visualized by Coomassie brilliant blue R-250 (CBB R-250) staining (Fig. 1B). Protein components were identified and labeled based on their migration on the SDS-PAGE system (5, 24). For both CpcG-PBS and CpcL-PBS, CpcA, CpcB, and all rod-linker proteins (e.g., CpcC1/2 and CpcG1/L) are present. There are also visual differences between the two PBSs. ApcE is absent from CpcL-PBS (Fig. 1B), consistent with a previous report (17). ApcA and ApcB (minor bands below CpcB and CpcA, respectively, in CpcG-PBS) also were not detected in CpcL-PBS. FNR_L_ is present in both CpcG-PBS and CpcL-PBS; however, CpcL-PBS contains a higher level of FNR_L_. CpcC2 protein content in CpcL-PBS also shows an increased level compared to that of CpcG-PBS, in contrast to a slightly decreased amount of CpcC1 (Fig. 1B).

### Quantitative mass spectrometry of CpcG-PBS and CpcL-PBS.

To quantify each protein component in the two PBS samples, CpcG-PBS and CpcL-PBS were subjected to trypsin digestion followed by LC-MS/MS, label-free protein quantification by using PEAKS Studio software (Bioinformatics Solutions Inc.) (25), and also manual quantification using extracted ion chromatograms assisted by Protein Metrics software (PMI). The heat maps of three technical replicates of protein profiles from the two types of PBSs indicate the compositional features (Fig. 1C). Protein sequence coverage of each identified subunit in both PBSs exceeds 80% (data not shown), indicative of effective LC-MS/MS sample preparation and analysis. This was achieved by using freshly prepared PBS samples. We detected no ApcA and ApcB in CpcL-PBS, resulting from genetic deletion in the mutant and consistent with a previous report (20). We also did not detect CpcG1, ApcC, ApcE, ApcF, and ApcD in CpcL-PBS (Fig. 1C), possibly because they tend to be degraded owing to the absence of ApcA and ApcB and consequently the failed assembly of a functional CpcG-PBS core or because they stay at the very top of the gradient. Notably, compared to CpcG-PBS, the observed level of FNR_L_ was consistently increased in CpcL-PBS in three independent biological replicates (Fig. 1C), and that same trend is followed for CpcC2 but not for CpcC1 (slightly decreased), another rod linker protein supposedly located on the proximal site of the CpcL protein. All these results are consistent with the general trends of the corresponding bands on SDS-PAGE (Fig. 1B). CpcG-PBS was successfully isolated and characterized in a mutant (*ΔcpcG2*) (17), while the isolation of CpcL-PBS was not successful by two independent laboratories (17, 20, 22). Our MS results unambiguously demonstrate that CpcG-PBS, free from contamination of CpcL (Fig. 1C), can be successfully isolated from WT cells as well as from *ΔcpcG2* mutant cells that were used for CpcG-PBS isolation (17). FNR_L_ was not previously reported in CpcL-PBS preparations (17, 20).

### Spectral characterization of CpcL-PBS.

To characterize further and compare the functional differences of the two PBSs, we obtained room temperature (RT) absorption spectra of CpcG-PBS and CpcL-PBS (Fig. 1D). The difference spectrum indicates the absence of allophycocyanin (APC) of the core, consistent with a previous study (20) and also with our SDS-PAGE (Fig. 1B) and LC-MS/MS results (Fig. 1C). The RT fluorescence emission spectrum with excitation at 580 nm shows two emission peaks at 646 nm and 667 nm, features that were previously reported but for a protein whose composition was unknown (4). The low-temperature (77 K) fluorescence spectrum shows a major emission, peaking at 669 nm with a small shoulder around 646 nm, indicative of efficient energy transfer to a pigment emitting at 669 nm. We name this red-shifted emission observed for CpcL-PBS “terminal energy emitter without allophycocyanin” (TEEWOAP).

### Subunit stoichiometry of linker proteins.

**FIG 2:**
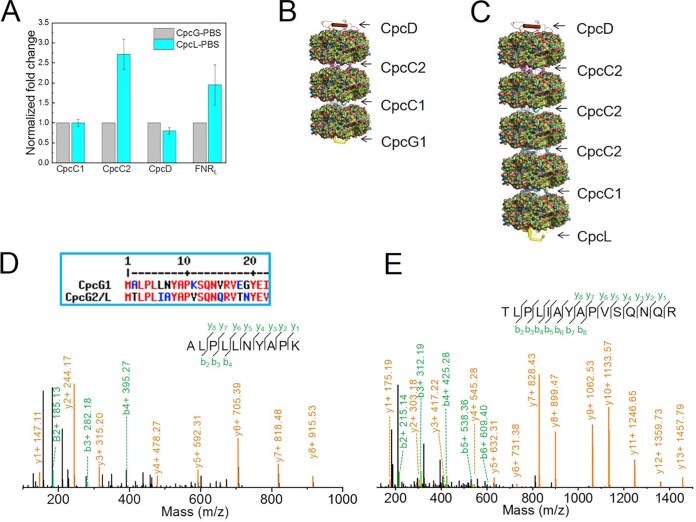
(A) Label-free rod polypeptide quantification of CpcG-PBS and CpcL-PBS. (Error bars indicate standard deviations.) (B and C) Model of a single rod of CpcG-PBS (B) and CpcL-PBS (C). (D) Product-ion (MS/MS) spectrum of CpcG1 protein N-terminal peptide. (Inset) N-terminal polypeptide sequence of CpcG1 (slr2051) and CpcL (sll1471) proteins. (E) Product-ion (MS/MS) spectrum of CpcL N-terminal peptide.

Label-free MS quantification using the PEAKS software package (25) gave significant differences between the two PBSs (Fig. 1C). The CpcL-PBS/CpcG-PBS ratios of CpcA, CpcB, CpcC1, CpcC2, FNR_L_, and CpcD are 0.99, 1.00, 0.82, 2.28, 1.99, and 0.77, respectively (Fig. 1C). Thus, CpcL-PBS has 2.8- and 2.4-fold higher content of CpcC2 and FNR_L_, respectively, than CpcG-PBS if CpcC1 is used as a normalization control, which is consistent with our SDS-PAGE analysis visualized by using CBB-R250 (Fig. 1B). To test further the results reached by using PEAKS software (see more information in Materials and Methods), we performed manual quantitative analysis assisted by the Protein Metrics software package (see more information in Materials and Methods) (Fig. 2A). Briefly, the peak area of the extracted ion chromatogram of a peptide from one sample in an LC-MS/MS experiment, in our case, CpcG-PBS, is computed and compared with that of the same peptide from another sample (i.e., CpcL-PBS) in a separate LC-MS/MS experiment of CpcL-PBS. Efforts were made to maximize the reproducibility of different LC-MS/MS runs. After normalization of the similar trend of CpcC1 and CpcD from CpcG-PBS and CpcL-PBS (see Materials and Methods), we found that the ratio of CpcC2 from CpcL-PBS and CpcG-PBS was 2.7:1, and the ratio of FNR_L_ was 1.9:1 (Fig. 2A). These results are consistent with and comparable to those calculated by using the PEAKS software.

Based on our biochemical (Fig. 1B) and two MS quantification methods, we conclude that the CpcC2 stoichiometry in CpcL-PBS is significantly different from that of the conventional CpcG-PBS rod, which contains 3 PC hexamers connected by one copy of CpcC1 and one copy of CpcC2 (14, 18, 26) (Fig. 2B). For the rod architecture of CpcL-PBS, there are 5 PC hexamers per rod, connected by one copy of CpcC1 on the proximal side of the CpcL protein and three copies of CpcC2 on the distal side of CpcL (Fig. 2C). The diffuse pattern of CpcL-PBS, however, in the sucrose density gradient separation indicates that CpcL-PBS is not homogenous and seems to support the hypothesis of various rod lengths but with an average of 5 PC per rod (Fig. 1A). Elucidating the biological significance requires further research.

Our MS data also reveal posttranslational modifications of both CpcG (CpcG1) and CpcL on their N termini. Figure 2D and E show the product-ion (MS/MS) spectra of the N-terminal peptides of CpcG1 and CpcL, respectively. MS/MS results indicate that the first methionine on both CpcG1 and CpcL was posttranslationally removed, leaving alanine (A) and threonine (T) as the first amino acid in CpcG1 and CpcL, respectively. CpcG1 and CpcL have molecular masses of 28.9 and 28.5 kDa, respectively, and pIs of 9.34 and 9.14, respectively. Although reports indicate that CpcG1 and CpcL can be effectively separated on SDS-PAGE (17, 22), our gel system did not unambiguously separate them (Fig. 1B). Nevertheless, our MS data (Fig. 1C and 2D and E) unambiguously confirm the identity of both proteins.

### FNR_L_ content heterogeneity in CpcL-PBS.

Although FNR_L_ was found in conventional PBS (i.e., CpcG-PBS [10, 13]), it has never been found in CpcL-PBS (4, 17). Previous studies reveal that conventional CpcG-PBS preparations contain an average of 1.3 FNR_L_ per PBS, with a maximum value of 2 per PBS (11). Thus, there is an average of 1.3 FNR_L_ per 6 rods, or 22% of CpcG-PBS rods contain FNR_L_. Our quantitative MS results indicate ∼2× larger amounts of FNR_L_ in CpcL-PBS (Fig. 1B and C and 2A), i.e., 44% of CpcL-PBS contains FNR_L_. That CpcL has a hydrophobic C-terminal domain, allowing enrichment in the Triton X-100 phase (22) of phase-partitioning experiments, does not mean that there is low abundance or negligible CpcL-PBS in WT cells. Rather, the abundance of CpcL protein was found to be ∼80% of CpcG1 in another quantitative proteomics study of WT *Synechocystis* 6803 cells (27). It should be noted that with progressive truncation of the CpcG-PBS rod (i.e., in a CpcC1/C2 double deletion mutant cell, called CB [5]), the abundance of the CpcL protein is close to or even higher than that of CpcG1 protein (27), indicating that the abundance of CpcG-PBS rod and CpcL-PBS are inversely related. We detected acetylation on the N terminus of FNR_L_ for both CpcG-PBS (6%) and CpcL-PBS (12%). Acetylation alters several protein properties, including molecular weight, stability, enzymatic stability, interactions with other proteins, and other biological functions (28, 29). Our results suggest that increased acetylation of FNR_L_ in CpcL-PBS has some biological significance, the analysis of which awaits future studies.

### Chemical cross-linking and identification.

**FIG 3:**
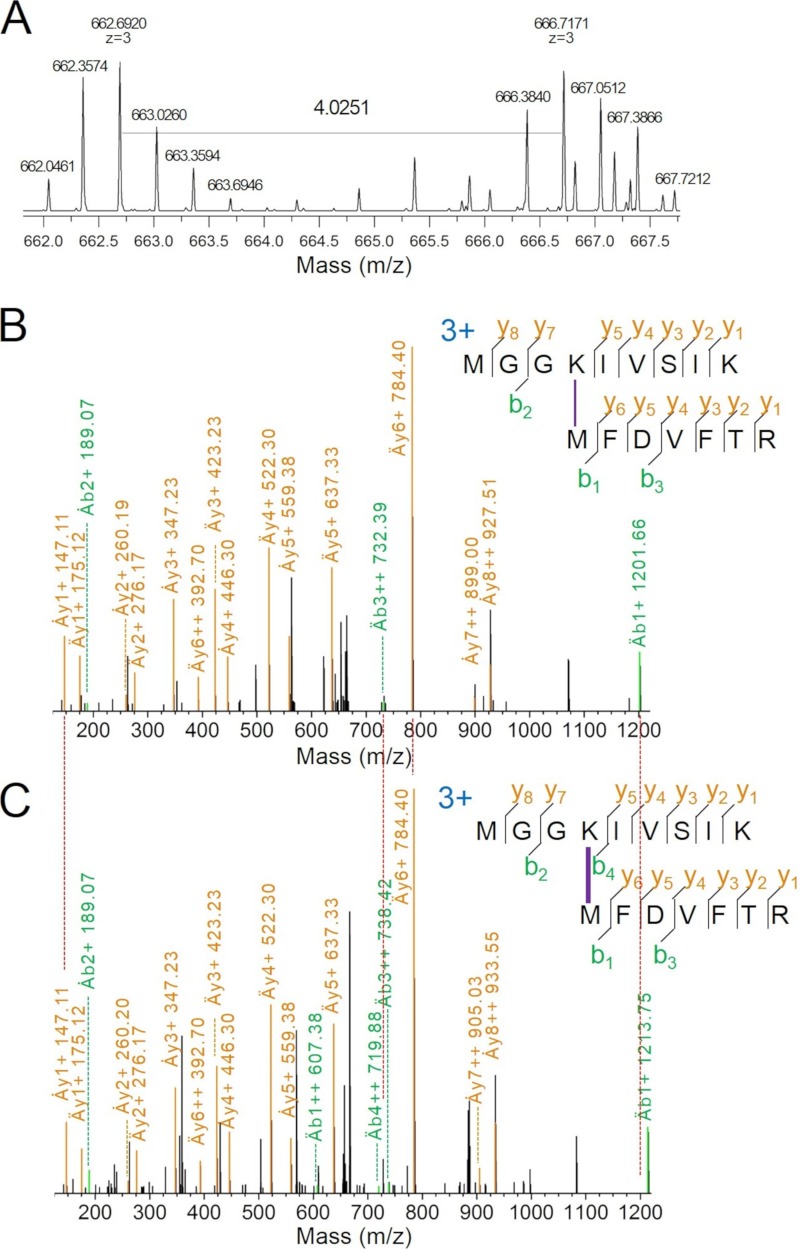
MS data showing a cross-link between FNR_L_-K69 and CpcB-M1 (N terminus). (A) The mass spectrum of precursors for the light and heavy cross-linked species (BS^3^-H_12_/D_12_), displaying the isotopic fingerprint of a peak doublet of equal intensity, separated by *m/z* 4.0251 (*z* = 3) (B) Product-ion spectrum of the cross-linked peptide with BS^3^-H_12_. (C) Product-ion spectrum of the cross-linked peptide with BS^3^-D_12_. Ȧ, top peptide; Ӓ, bottom peptide.

The structural location of FNR_L_ in both PBSs is of crucial interest. We subjected both PBS samples to chemical cross-linking with a 1:1 mixture of BS^3^ (bis[sulfosuccinimidyl]suberate) (H_12_/D_12_) cross-linker (30) and analyzed the results by LC-MS/MS. It should be noted that the yield of chemical cross-linking is usually very low. Only when two reactive groups, i.e., sulfo-NHS ester used in this study, react with primary amine-containing molecules from two different peptides can they produce useful information for elucidating two proteins’ structural proximity. In practice, the outcome of protein cross-linking reaction is affected by solvent accessibility of the primary amine and distance between two functional groups. In our experiments, we detected many monolinks (36 in CpcL-PBS and 75 in CpcG-PBS) on peptides that are modified once by the cross-linker but are not linked to a second peptide, probably because a second functional group to complete the cross-link is not readily available (data not shown). In addition to many monolinks, we also identified many loop links and cross-links that are on other PBS subunits. We identified three interprotein cross-links connecting FNR_L_ and either CpcB or CpcA. Figure 3 shows one cross-link of two peptides from FNR_L_ and CpcB: FNR_L_ (MGGK^69^IVSIK) and CpcB (^1^MFDVFTR, N-terminal primary amine of methionine). The precursor ions of this cross-link appear as a doublet (1:1) owing to isotope coding on the cross-linkers (i.e., from light BS^3^-H_12_ and heavy BS^3^-D_12_ [Fig. 3A]). The doublet peaks show an *m/z* 4.0251 shift. Because the charge state is 3+, the mass difference is 12.0753 (3 × 4.0251), exactly as expected from the mass difference between BS^3^-H_12_ and BS^3^-D_12_. Overall, the product-ion coverage is 93% for y ions and 21% for b ions. The isotopic ion coverage (both y and b ions) is 29%. All major fragments (y and b) in the product-ion spectra match predicted peptide fragments, resulting in a highly confident identification (Fig. 3B and C).

**FIG 4:**
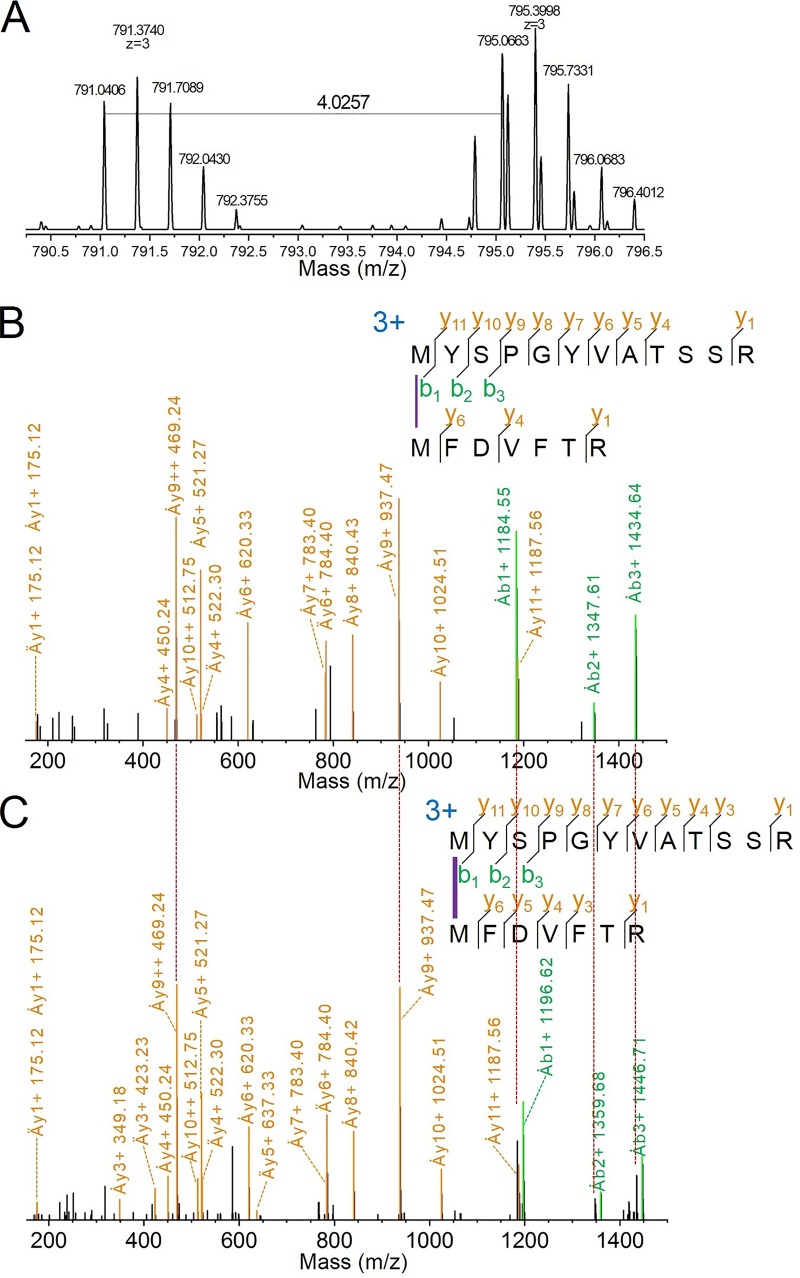
MS data showing a cross-link between FNR_L_ and CpcB. Reference ions without the cross-linker reagent (Ȧy9++ at *m/z* 469.24, Ȧy9+ at *m/z* 937.47) and ions with the characteristic 12-Da shift that contain cross-linkers are indicated (Ȧb1+, Ȧb2+, and Ȧb3+). Overall, the product-ion coverage is 71% for y ions and 18% for b ions. The isotopic ion coverage (both y and b ions) is 18%.

We also identified another cross-link between FNR_L_-^1^MYSPGYVATSS and CpcB-^1^MFDVFTR (Fig. 4A to C). The precursor ion mass spectra and the two product-ion (MS/MS) spectra containing light and heavy cross-linkers (Fig. 4B and C) lend high confidence to the assignment.

Using two protein cross-linking search engines, we identified two cross-links (Xlinks) between FNR_L_ and CpcB (Xlink1 and Xlink2) and one cross-link between FNR_L_ and CpcA (Xlink3) (Table 1). The amino acid residues joined in the cross-linking are FNR_L_:K^69^-CpcB:^1^M, FNR_L_:^1^M-CpcB:^1^M, and FNR_L_:^1^M-CpcA:^1^M. The criteria used for our identification are stringent, in accordance with literature requirements (31, 32).

**TABLE 1:** Spatial distance analysis of cross-linked pairs using Xwalk^*a*^

Model and Xlink	FNR_L_	CpcB	CpcA	Distance (Å)			
				Euclidean	Amine	SASD	RMSD
A							
1	K69	M1		13.9	11.2	23.8	19.5
2	M1	M1		24.8	23.5	33.1	
3	M1		M1	30	27	34.2	
B							
1	K69	M1		13.6	8.4	22.3	25.9
2	M1	M1		27.2	24.8	44.3	
3	M1		M1	32	29.8	39.9	
C							
1	K69	M1		14.4	9.4	24.1	23.9
2	M1	M1		25	25.6	40.3	
3	M1		M1	28.8	28.7	38.3	
D							
1	K69	M1		14.6	13.5	19.9	20.7
2	M1	M1		24.1	21.3	36.1	
3	M1		M1	28.5	25	37.2	
E							
1	K69	M1		14.1	9.9	21.2	10.7
2	M1	M1		15.3	13.8	22.2	
3	M1		M1	18.4	16.4	23.1	

a Xwalk (spatial distance) analysis (51) of cross-linking pairs from Fig. 6A. Listed are the Euclidean Cα-Cα distances, the side chain amine groups distances, and SASD (see the text for details) between paired amino acids. X_i_, Xwalk calculated value (Å). C = 11.4 Å. RMSD was calculated as $\sqrt{\frac{1}{n}\overset{n}{\underset{i=1}{\sum }}{\left({\text{X}}_{\text{i}}-c\right)}^{2}}$ .

### Modeling: strength and limitations.

**FIG 5:**
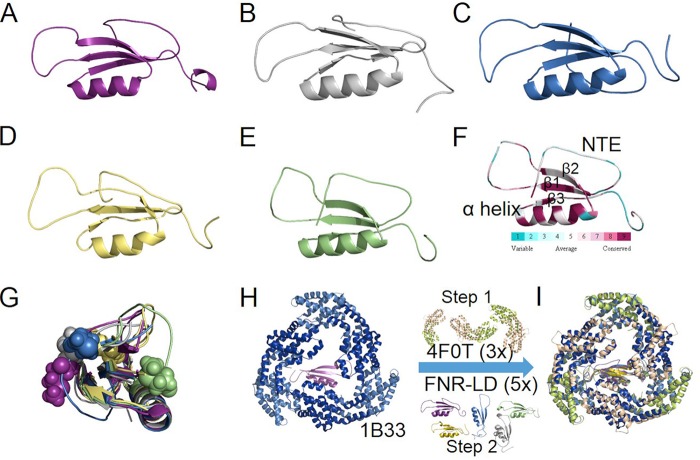
Iterative threading assembly refinement (I-TASSER) method for protein structure prediction of FNR_L_ N-terminal linker domain (FNR_L_-LD; 89 amino acids). (A to E) Five models are shown with decreasing C-scores (I-TASSER). (F) Bioinformatics analysis of FNR_L_-LD using the ConSurf server (38, 39, 53), showing the conserved (purple) α helix and β sheet and the variable loop regions and N-terminal flexible domain (teal). (G) Side views of the five models of FNR_L_-LD. The first methionine of each model is shown as a sphere with color rendering consistent with models in Fig. 1A to E, respectively. (H) Cartoon representation of allophycocyanin core linker (ApcC) complex: ApcA (marine), ApcB (dark blue), and ApcC (purple). (I) Three rounds of alignment of CpcA/B heterodimer (*Synechocystis* 6803 α and β subunit; PDB entry 4F0T) with ApcA/B (1B33), followed by homology modeling of five predicted FNR_L_-LD structures with ApcC (PDB entry 1B33).

Using I-TASSER, we generated five models of FNR_L_-linker domain (LD) (Fig. 5A to E). Protein structure prediction was performed in the Zhang Server by using a template of PDB entry 1B33 (33) without any additional restraints as guides (34–36). FNR_L_-LD belongs to the CpcD superfamily (cl03191) of proteins (or rod-capping linker) that are involved in assembly of the phycobilisome (37). The X-ray crystallographic structure of an electrophoretically purified allophycocyanin linker complex (trimeric APC-L_C_^7.8^; PDB entry 1B33) (33) serves as a reference. ApcC (L_C_^7.8^), a CpcD superfamily protein, was resolved in the structure and consequently was used as the default template for the homology-modeling algorithm by I-TASSER. Each of the five models has an elongated shape and consists of a three-stranded β sheet (β1, F^21^-I^26^; β2, S^46^-V^51^; β3, K^69^-V^71^) and one or two α-helices, accounting for 48% of the secondary structure and 52% of the loop region (Fig. 5F).

### Bridging modeling and chemical cross-linking data.

Using the ConSurf Server (38, 39), we identified the evolutionarily conserved and variable amino acids (Fig. 4F). FNR_L_-LD contains an N-terminal extension (NTE; M^1^-N^18^) and a loop region connecting β-1 and β-2 (S^29^-P^40^). In all five models, the α-helix (L^53^-R^65^) is always located on one side of a *β*-sheet (Fig. 5A to F). CpcD and FNR_L_-LD have a longer NTE and no template for structural prediction (data not shown). In the predicted models, orientation of the NTE relative to the conserved secondary structures is variable and is located either on the same side of the α-helix (model E) or on the opposite side (models A, B, C, and D) (Fig. 5G), leading us to wonder what methodology is appropriate to constrain the *in silico* models, as in this case, when no structures exist of isolated FNR_L_ and CpcD family proteins.

Our modeling is aimed at achieving a trimeric CpcA/B-FNR_L_-LD complex by using PDB entry 1B33 as a general template for heterodimeric CpcA/B and FNR_L_-LD (Fig. 4H and I). We performed three rounds of CpcA/B modeling of the *Synechocystis* 6803 CpcA/B (αβ) crystal structure (PDB entry 4F0T) (40) and five rounds of FNR_L_-LD (Fig. 5A to E) modeling. One issue is the location of the NTE relative to other secondary structures (β-sheet and α-helix) within FNR_L_-LD and relative to its secondary structure and the three heterodimeric CpcA/B in the context of the CpcA/B-FNR_L_-LD complex.

**FIG 6:**
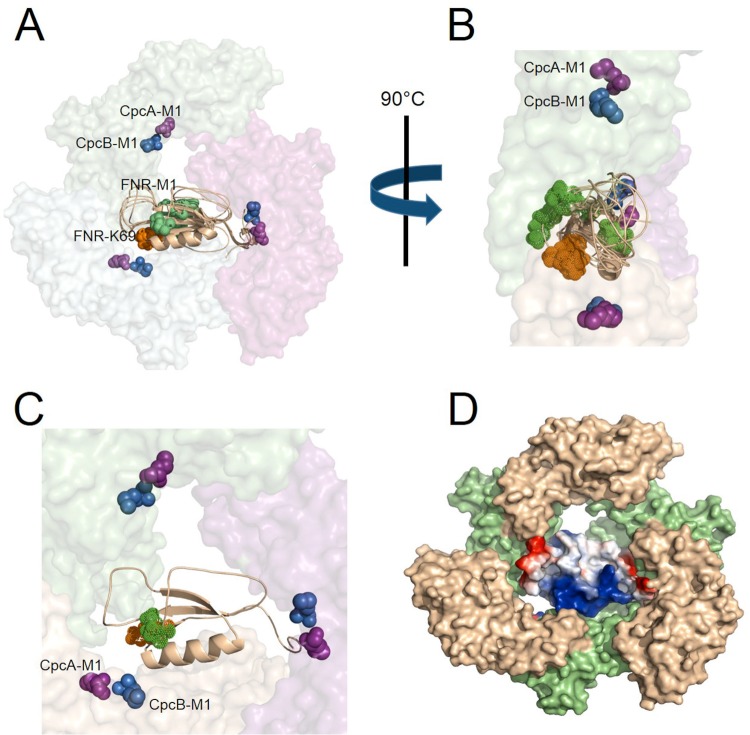
(A) View of all amino acids involved in FNR_L_-LD-PC cross-linking: CpcB-1M (purple), CpcA-1M (blue) from trimeric phycocyanin, K69 (orange), and 1M (green) of FNR_L_. K69 and 1M from five models are shown. (B) Side view of FNR_L_-LD (5 models) in PC trimer, highlighting K69 and 1M locations relative to the α-helix and β-sheet of FNR_L_-LD and the PC trimer. (C) Optimized model (model E in Fig. 5B) with the least spatial conflicts and most favorable cross-linking chemistry. The N-terminal extension region of FNR_L_ adopts an orientation close to the proximal side of CpcA. K69 and M1 of FNR_L_ are located close to N termini of CpcA and CpcB in one heterodimer. (D) Surface representation of trimeric phycocyanin and electrostatic potential surface representation of FNR_L_-LD. CpcA, wheat; CpcB, lime.

For a specific model of an FNR_L_-LD associated with trimeric CpcA/B (Fig. 5I), the distances of each cross-link pair can be used to adjudicate the model (Fig. 6A and B). Similar to ApcA/B-L_C_^7.8^ (PDB entry 1B33), our trimeric Cpc-FNR_L_-LD model has the CpcA/B:FNR_L_-LD stoichiometry of 3:1. This means that, given the observed cross-link FNR_L_:K^69^-CpcB:^1^M, CpcB:^1^M can involve any methionine of the trimeric CpcB (Fig. 6A and B). In Table 1, the distance for each cross-link pair represents the smallest of the three. For each nearest cross-linked pair, three spatial distance metrics can be mapped: Euclidean Cα-Cα, distance between two primary amine groups (N_ζ_), and solvent-accessible surface distance (SASD) (Table 1).

Upon visual check of all the values (Table 1), we conclude that the values of Xlink 1 from all five models are consistent with the cross-linking results (41–43) on the basis of the measured Euclidean distances, amine, and SASD (top row of each model). Here, we introduce another metric to evaluate globally the structural consistency of all three cross-links, namely root-mean-square deviation (RMSD) (Table 1). The smaller the RMSD, the more likely the cross-linking chemistry can occur and consequently the better the model fits the experimental data. Among the five models, model E has the lowest RMSD value, at 10.7 Å (Table 1). The FNR_L_-LD model E in trimeric PC (Fig. 6C) shows the NTE is on the same side as the α-helix rather than the other side of the β-sheet (Fig. 6D). This model also locates the NTE in the central region if two trimeric PCs form a hexamer.

Previous reports indicate the conventional CpcG-PBS contains an average of 1.3 FNR_L_ per PBS (11). We identified identical cross-linking species from both CpcG-PBS and CpcL-PBS, indicative of the similar structural location of FNR_L_ in both types of PBS. The reasons for the increased level of FNR_L_ observed in CpcL-PBS and its functional significance await future research. Previous reports also indicate that CpcL-PBS preferentially transfers energy to photosystem I (18, 22). Indeed, CpcL-PBS (or CpcG2-PBS) forms an NDH-1-CpcG2-PSI supercomplex that facilitates PSI cyclic electron transport via NDH-1 instead of its involvement in respiration (44). Our discoveries of a *bona fide* association of FNR_L_ and CpcL-PBS and the increased level of FNR_L_ in CpcL-PBS may shed new light on the diversified energy conversion strategies in cyanobacteria, namely, photosystem I photochemistry, ferredoxin and NADP^+^ oxidoreduction, and cyclic electron transfer in a supercomplex. FNR_L_ is not identified in the recently solved PBS structure from the red alga Griffithsia pacifica (3), indicating that FNR_L_ binding to PBSs is not a characteristic for all PBS-retaining oxygenic photosynthetic organisms (45). CpcL-PBS, heterogeneous in length and FNR_L_ content, and their distribution across heterogeneous electron transport machinery in different thylakoid regions or patches are subjects for future research.

## MATERIALS AND METHODS

### Growth of *Synechocystis* sp. strain PCC 6803 and PBS isolation and characterization.

Cyanobacterial strains were grown in BG-11 medium supplemented with 20 mM TES (2-[(2-hydroxy-1,1-bis(hydroxymethyl)ethyl)amino]ethanesulfonic acid, *N*-[tris(hydroxymethyl)methyl]-2-aminoethanesulfonic acid)-KOH (pH 7.5) at 30°C. Liquid cultures were grown in a 10-liter carboy with fluorescence illumination from both sides, light intensity of 50 µmol photons m^−2^ · s^−1^, and air bubbling from the top and magnetic bar stirring at the bottom driven by a stir plate underneath the carboy. Cell cultures were harvested at log growth phase at an optical density at 730 nm of 0.2 to 0.3 and resuspended in 0.8 M K-phosphate buffer. CpcG-PBS and CpcL-PBS were isolated from CpcL-PBS (WT) and the ΔAB mutant, which contains no ApcA/B due to the genetic deletion of the *apcABC* operon (20) (the latter was a generous gift from Ghada Ajlani. Briefly, the cells supplemented with protease inhibitor cocktail (Thermo Fisher Scientific, Waltham, MA, USA) and DNase (Sigma, St. Louis, MO) were broken in 0.8 M K-phosphate buffer at pH 7.5 by passing through three rounds of a French press (prechilled at 4°C) at 1,500 lb/in^2^. After 0.5 h of incubation with 2% Triton X-100 (Sigma, St. Louis, MO, USA) at room temperature, the blue liquid supernatant was loaded immediately onto a sucrose gradient (46). After ultracentrifugation (370,000 × *g*) overnight, blue bands of CpcG-PBS and CpcL-PBS samples were collected at the interface of 0.75 M to 1.0 M sucrose and the 0.75 M sucrose region, respectively, and analyzed by using SDS-PAGE. PBS samples without cross-linking chemistry were also directly subjected to trypsin digestion and LC-MS/MS analysis for PBS subunit identification and quantification.

### Protein cross-linking.

The isotope-coded cross-linking experiment was performed as previously described, with minor changes (30, 47). Briefly, PBS was diluted and resuspended at 0.1 µM in 0.2 M K-phosphate buffer supplemented with a 1:1 mixture of unlabeled BS^3^ and BS^3^ labeled with 12 deuterium atoms (BS^3^-H_12_/D_12_; Creative Molecules, Inc.) for 10 min in the dark at room temperature at a cross-linker/PBS molar ratio of 10:1, 50:1, and 100:1. Quenching and desalting of PBS samples were achieved by using Zeba spin columns (Thermo Fisher Scientific, Waltham, MA, USA).

### Protein sample digestion, LC-MS/MS, and quantitative MS.

Desalted cross-linked PBS samples, as well as untreated PBS samples, were precipitated by adding acetone and digested with lysyl endopeptidase (LysC) and then trypsin by following a previously published method (48). Briefly, protein pellets were dissolved in an 8 M urea solution (20 µl) followed by incubation with tris(2-carboxyethyl)phosphine (2.5 mM) at 37°C for 30 min and treated with iodoacetamide (5 mM) for 30 min at room temperature. After LysC digestion (0.05 µg/µl) for 2 h followed by 8× dilution, the protein solution was further incubated with trypsin overnight at 37°C. Finally, the digestion was quenched by adding 0.1% formic acid. Aliquots (5 µl) of the peptide samples were analyzed with a Q-Exactive Plus mass spectrometer (Thermo Fisher Scientific, Waltham, MA, USA) operated in standard data-dependent acquisition mode controlled by Xcalibur, version 3.0.63. Calibrations of the mass spectrometer followed the manufacturer’s protocol. Precursor activation was by HCD (higher-energy collision-induced dissociation), which was set with an isolation width of *m/z* 1.5 and a normalized collision energy of 27%. The mass resolving power employed was 70 K for precursor ions and 17.5 K for product ions (MS2).

The raw data were loaded into PEAKS (version 8.5; Bioinformatics Solution, Inc., Waterloo, ON, Canada) for protein identification and label-free quantification (25). The data were searched against a database of the *Synechocystis* phycobilisome proteins by using the built-in fusion decoy database for false discovery rate calculation. Search parameters were the following: precursor ion mass tolerance, 10.0 ppm; fragment ion mass tolerance, 0.02 Da; variable modifications, all built-in modifications plus the user-defined light and heavy monolink forms of the cross-linker, 156.0079 and 168.1540 Da, respectively. The instrument mass resolving power was higher than that suggested for reliable cross-linking (31): maximum variable modifications per peptide, 3; maximum missed cleavages, 2; maximum nonspecific cleavages, 0; false discovery rate, 0.1%. Label-free quantification was performed by using the built-in protocol in PEAKS, with the final heatmap in Fig. 1C reflecting a comparison of three replicates of each type of PBS sample. For technical details, see reference 30.

For the second round of label-free quantification, the LC-MS/MS raw data were submitted to Protein Metrics software (PMI). Database searching was performed by Byonic software using the *Synechocystis* 6803 phycobilisome protein database and a decoy database containing reversed protein sequences. Search parameters were the following: precursor ion mass tolerance, 20 ppm; fragment ion mass tolerance, 60 ppm; maximum missed cleavage, 2. Common posttranslational modifications and the automatic peptide score were cut. The protein false discovery rate threshold was determined by the score of the highest-ranked decoy protein identified. The search results were combined in Byologic software for validation and extraction of ion chromatograms with a mass window of 20 ppm. Peptides used for protein quantification must meet the following criteria: fragment ion spectrum score above 300, correct monoisotopic mass, and low relative intensity (below 10%) of interference peaks within the peptide isotopic pattern. Chromatogram peaks with multiple MS/MS identifications were integrated by the software for quantification of that peptide. For each protein, at least three peptides were used for relative quantification of that protein. Cpcc1 and CpcD were used for normalization because they are in a 1:1 ratio in the CpcG-PBS. We chose 19 of 25 peptides of FNR_L_ from each PBS for consideration.

### Identification of cross-linked peptides.

ICC-Class (49, 50) and pLink software (32, 51) were used for identification of cross-linked peptides. For ICC-CLASS, the “mgf-only” DXMSMSMatch program was used, and settings were the following: cross-linker, DSS; DX, 12.07532; DX mass tolerance, 0.013 Da; DX retention time tolerance, 60 s; filter DX mass tolerance, 5 ppm; filter DX time window, 60 s; digest sites, KR, including cross-link sites; missed digest sites, up to 4; cross-link site, K (N-terminal primary amine included); precursor tolerance, 5 ppm; fragment tolerance, 30 ppm. Candidate cross-linked peptides were inspected manually to verify the assignment.

The raw LC-MS/MS files were also analyzed by pLink (ver. 2.3.1; Institute of Computing Technology, Chinese Academy of Sciences, Beijing, China). The PBS protein sequence was added manually into the search database. Cross-linker information, including monoisotopic linker masses of light and heavy forms of BS^3^ (156.079 Da and 168.154 Da, respectively), linked sites, and composition were all required in a configuration file (pConfig). pLink was implemented with the following settings: enzyme, trypsin and up to three missed cleavages; precursor tolerance, 20 ppm; fragment tolerance, 60 ppm; variable modifications, oxidation of M, deamidation of N, Q, and N terminus; minimal peptide length, 6 amino acids; maximal peptide length, 60 amino acids; minimal peptide mass, 600 Da; maximal peptide mass, 6,000 Da. The cross-linked peptides were examined with pLabel, affording a corresponding summary report. The cross-links were identified at a false discovery rate equal to or smaller than 5% at spectral level with a 10-ppm filter tolerance. Isotopic doublets signifying a likely cross-link were manually confirmed in the raw file. Theoretical product-ion information was generated through Protein Prospector MS-Product (http://prospector.ucsf.edu/) for the cross-linked peptides that are shown in the pLink search. Following that, likely relevant product-ion spectra were further validated manually.

### Protein structure prediction and modeling.

I-TASSER or Zhang Server was used for FNR_L_-LD (N-terminal 89 amino acid of FNR_L_; *slr1643*) and CpcD (*ssl3093*) protein structure prediction (34, 35, 52).
